# Associations between weight loss history and factors related to type 2 diabetes risk in the Stop Diabetes study

**DOI:** 10.1038/s41366-021-01061-4

**Published:** 2022-01-12

**Authors:** F. Halali, A. Lapveteläinen, K. Aittola, R. Männikkö, T. Tilles-Tirkkonen, E. Järvelä-Reijonen, P. Absetz, M. Kolehmainen, U. Schwab, J. Lindström, T. A. Lakka, J. Pihlajamäki, L. Karhunen

**Affiliations:** 1 School of Medicine, Institute of Public Health and Clinical Nutrition, University of Eastern Finland, Kuopio, Finland; 2 Health Sciences Unit, Faculty of Social Sciences, Tampere University, Tampere, Finland; 3 Collaborative Care Systems Finland, Helsinki, Finland; 4 Department of Medicine, Endocrinology and Clinical Nutrition, Kuopio University Hospital, Kuopio, Finland; 5 Department of Public Health and Welfare, Finnish Institute for Health and Welfare, Helsinki, Finland; 6 Institute of Biomedicine, School of Medicine, University of Eastern Finland, Kuopio, Finland; 7 Department of Clinical Physiology and Nuclear Medicine, Kuopio University Hospital, Kuopio, Finland; 8 Foundation for Research in Health Exercise and Nutrition, Kuopio Research Institute of Exercise Medicine, Kuopio, Finland

**Keywords:** Nutrition, Weight management

## Abstract

**Background:**

Frequent weight loss attempts are related to maladaptive eating behaviours and higher body mass index (BMI). We studied associations of several type 2 diabetes (T2D) risk factors with weight loss history, defined as the frequency of prior weight loss attempts, among Finnish adults at increased risk for T2D.

**Methods:**

This study (*n* = 2684, 80% women) is a secondary analysis of the 1-year StopDia lifestyle intervention with digital intervention group, digital intervention + face-to-face counselling group, or control group. The frequency of prior weight loss attempts was categorized into five groups: no attempts/no attempts to lose weight, but trying to keep weight stable/1–2 attempts/3 or more attempts/ continuous attempts. Data on emotional eating and social/emotional nutrition self-efficacy were collected with a digital questionnaire. We assessed baseline differences between categories of weight loss history as well as the intervention effects.

**Results:**

Altogether 84% of participants had attempted weight loss. Those with one or more weight loss attempts had higher BMI, larger waist circumference, and more emotional eating compared to ‘no attempts’ and ‘no attempts to lose weight, but trying to keep weight stable’ categories. The ‘no attempts’ category had the highest baseline fasting insulin, whereas it showed the largest decrease in this measure with the intervention. This change in fasting insulin in the ‘no attempts’ category was significantly different from all the other categories. Emotional nutrition self-efficacy slightly improved in the ‘no attempts’ category, which was significantly different from its concomitant decrease in the categories ‘1-2 attempts’ and ‘3 or more attempts’. The intervention group assignment did not affect the results.

**Conclusions:**

Multiple attempts to lose weight may unfavourably affect T2D risk factors as well as lifestyle intervention outcomes. More research is needed on how weight loss frequency could affect T2D risk factors and how to design lifestyle interventions for individuals with frequent previous weight loss attempts.

## Introduction

Type 2 diabetes (T2D) is a continuously growing global health problem [1]. An increase in the prevalence of T2D in parallel with the increase in obesity prevalence mirrors their close relationship, as obesity is associated with more cardiovascular disease outcomes, including a significantly higher risk of T2D incidence [2]. Interventions focusing on the two major lifestyle factors, diet and physical activity, have proven successful in preventing T2D among those at high risk for the disease [3, 4].

Dieting and attempting to lose weight have become increasingly common among the general population while maintaining the lost weight remains the main challenge [5–7]. On the other hand, the greater number of attempts to lose weight and weight instability have been associated with higher body mass index (BMI) [8–11], higher prevalence of binge eating disorder in women with obesity [12] as well as in both sexes in a general population [13], greater eating disinhibition in women [13] and maladaptive eating behaviours [14], all of which could mediate the risk of developing T2D. One type of maladaptive eating behaviours associated with weight loss attempts and dieting is emotional eating, which is eating in response to negative emotions [15]. Emotional eating has also been associated with higher BMI [16], greater consumption of energy-dense, high-fat foods [17], poorer weight loss maintenance [18], as well as a higher likelihood of central obesity and T2D [19].

Repeated failed attempts to lose weight could lead to decreased self-efficacy [20], i.e. lower confidence in one’s ability to make changes according to desired goals [21]. Nutrition self-efficacy is one of the psychosocial determinants of adopting healthy dietary behaviours and weight loss [22–24]. Some studies have reported that better dietary self-efficacy is associated with more consumption of fruits and vegetables [25, 26], as well as with reduced fat intake and improved glycemic control in adults with T2D [27, 28], although this has not been confirmed in all studies [29].

Given the ever-growing burden of T2D along with ongoing efforts to lose weight, we studied the associations of anthropometric, metabolic, psychological and lifestyle factors with weight loss history, defined as number of prior attempts to lose weight, at baseline and at the end of a 1-year lifestyle intervention, among Finnish adults identified to be at increased risk for T2D.

## Materials and methods

### Study design and participants

The present study is a secondary analysis of Finnish adults with an increased risk for T2D who participated in the 1-year randomized controlled trial (RCT) of Stop Diabetes (StopDia) (ClinicalTrials.gov registration no. NCT03156478). General aim of the StopDia was to create and implement evidence-based and digitally supported strategies for the prevention of T2D at population level. Details of the StopDia study have been published elsewhere [30]. Briefly, 3265 participants were recruited to participate in the study using the StopDia digital screening tool, including questions from the Finnish Diabetes Risk Score (FINDRISC) questionnaire [31] as well as other questions on inclusion and exclusion criteria for RCT [30]. Participants to take part in the RCT were those who (i) had FINDRISC ≥ 12 points or previous gestational diabetes or history of impaired fasting glucose (IFG) or impaired glucose tolerance (IGT); (ii) were residing in one of the three Finnish provinces (Northern Savo, Southern Carelia, or Päijät-Häme); (iii) had possibility to use a computer, smartphone or tablet with an internet connection; (iv) had a personal cell phone number and (v) had adequate Finnish language skills. The exclusion criteria were (i) diagnosed type 1 or type 2 diabetes; (ii) pregnancy or breastfeeding and (iii) current cancer or less than 6 months since the last cancer treatment. Eligible participants visited a study nurse in their local health care center for baseline anthropometric measurements and reference for clinical measurements in the local laboratories. At this stage, participants signed a written informed consent form. Participants who subsequently gave blood samples for laboratory measurements and filled out the web-based StopDia digital questionnaire were randomly assigned into one of the three RCT groups: (i) digital lifestyle intervention group, in which participants received lifestyle intervention through the ‘BitHabit’ web application, (ii) digital lifestyle intervention and face-to-face counselling group, in which participants received lifestyle intervention through the ‘BitHabit’ web application and six face-to-face group counselling sessions and (iii) control group, in which participants merely received written information on healthy diet and physical activity. Diet and physical activity-related goals in the intervention groups (i) and (ii) as well as content of the written information provided to the control group were built upon established recommendations of Nordic Nutrition Recommendations [32], Finnish Nutrition Recommendations [33] and international physical activity guidelines [34–36].

For the present study, we selected those who had participated in the RCT based on having FINDRISC ≥ 12 points, previous gestational diabetes, or confirmed IFG/IGT by the StopDia laboratory measurements. In addition, those who had current eating disorders were excluded (*n* = 23). The final number of participants in the present study was 2684 (80% women).

Study data were collected and managed using REDCap electronic data capture tools hosted at the University of Eastern Finland [37, 38]. REDCap (Research Electronic Data Capture) is a secure, web-based software platform designed to support data capture for research studies, providing (1) an intuitive interface for validated data capture; (2) audit trails for tracking data manipulation and export procedures; (3) automated export procedures for seamless data downloads to common statistical packages and (4) procedures for data integration and interoperability with external sources.

### Measures

#### FINDRISC questionnaire

The FINDRISC questionnaire is a practical tool to screen for the risk of developing T2D within the next 10 years of life [31]. It consists of eight questions about age, BMI, waist circumference, weekly physical activity, daily consumption of vegetables, fruit or berries, use of anti-hypertensive medication, history of having high blood glucose concentrations, and family history of diabetes. The total FINDRISC score is the sum of scores of its questions and it ranges from 0 to 26. The higher the FINDRISC score the higher the risk of developing T2D during the next 10 years.

#### Background characteristics

For background information, the StopDia digital questionnaire included questions about individuals’ sociodemographic characteristics (e.g. age, marital status, education level), history of diagnosed non-communicable diseases (e.g. coronary heart disease) and history of medication (e.g. use of anti-hypertensive medication).

#### Lifetime history of weight loss

Individuals’ weight loss history was assessed within the StopDia digital questionnaire by asking: Have you tried to lose weight during your lifetime? The question had five answer options: (i) No, (ii) No, but I have been trying to keep my weight stable, (iii) Yes, 1–2 times, (iv) Yes, ≥3 times, (v) Yes, continuously [39].

#### Anthropometric, clinical and biochemical measures

Trained nurses performed the measurements at the local health care centers and the local laboratories. Body weight was measured in light clothing by digital scales to accuracy of 0.1 kg. Height was measured in a Frankfurt position and without shoes to accuracy of 1 cm. Waist circumference was measured in standing position on bare skin at the end of normal exhalation and at the mid-distance between the bottom of the rib cage and the top of the iliac crest to accuracy of 1 cm. Following a 5-minute rest, resting blood pressure was measured twice, with a 2-min interval, from the right arm in a sitting position to accuracy of 1 mmHg, and mean of the two measurements was reported for systolic and diastolic blood pressures.

After a 12 hour overnight fasting, blood samples were taken to be analysed for glucose and insulin concentrations [fasting and 2 hours after the ingestion of 75 g glucose in an oral glucose tolerance test, glycated haemoglobin (HbA1c)] and fasting plasma total and lipoprotein lipid concentrations [total cholesterol, low-density lipoprotein (LDL) cholesterol, high-density lipoprotein (HDL) cholesterol and total triglycerides]. The disposition index, as a measure of early-phase insulin secretion, and the Matsuda index, as a measure of peripheral insulin sensitivity, were calculated using commonly used formulas [30, 40, 41].

#### Psychobehavioural measures

The StopDia digital questionnaire included measures of emotional eating and nutrition self-efficacy. Emotional eating was measured using the three items of the Three-Factor Eating Questionnaire (TFEQ-R18) [42] [*When I feel anxious, I find myself eating; When I feel blue, I often overeat; When I feel lonely, I console myself by eating*]. These items are scored on a 4-point scale with a higher score indicating a higher level of emotional eating. The final score is the sum of the raw scores of the three items transformed into a 0–100 scale [((raw score-lowest possible raw score)/possible raw score range)*100] [43]. Nutrition self-efficacy was assessed using a 10-item questionnaire [44, 45]. The general stem for all items was *How certain are you that you could overcome the following barriers?* [23], followed by five items measuring social nutrition self-efficacy (the belief in being able to deviate from the social norms), and five items measuring emotional nutrition self-efficacy (the belief in being able to stick to healthy eating in negative psychological states). The item scores range from 1 (very certain I cannot) to 4 (very certain I can) [46], and the final score is calculated as the mean of the related items. A higher score indicates a higher level of total/social/emotional nutrition self-efficacy.

#### Dietary measures

Dietary intake was measured using an 18-item food intake questionnaire within the StopDia digital questionnaire. Healthy Diet Index (HDI), which has been developed and validated based on this 18-item questionnaire for assessing diet quality as compared to the Nordic Nutrition Recommendations was computed [32, 47]. HDI consists of seven weighted domains: meal pattern, grains, fruit and vegetables, fats, fish and meat, dairy, and snacks and treats. The total HDI score gives a comprehensive measure for diet quality based on the sum score of these domains. It ranges from 0 to 100, with a higher score indicating a better diet quality [47].

### Data analysis

Statistical analyses were performed with the SPSS statistical software, version 27 (IBM SPSS Statistics for Windows, Armonk, NY). Differences and associations with *P*-values ≤ 0.05 were considered statistically significant. Continuous variables are reported as means (SDs) and categorical variables are reported as frequencies (percentages). For assessing baseline differences in the outcomes between the categories of weight loss history, we used analysis of covariance (ANCOVA) for continuous variables, and Chi-squared test for categorical variables. The ANCOVA analysis for BMI was adjusted for age and sex. The analyses for other outcome measures were adjusted for age, sex and BMI. The analyses for blood pressure measures were additionally adjusted for taking blood pressure medication and those of plasma lipid measures were adjusted for taking lipid-lowering medication.

In order to study how weight loss history contributed to changes in outcome measures during 1 year of intervention, we tested for the ‘lifetime history of weight loss × time’ interaction using the intention-to-treat principle, including all 2684 participants in the analyses. Linear mixed-effect model analysis was used, and for each of the outcome measures we tested several models with allowing/ignoring random intercept and random slope on subject and/or study province level [48]. The Bayesian information criterion (BIC) was used as a measure of model adequacy [49, 50]. We selected the model with the lowest BIC value as the best-fitting model for a given outcome variable. We also included in all the analyses the three-way interaction of ‘lifetime history of weight loss × time × intervention group’ to see if 1-year changes in outcome measures in categories of weight loss history were different between the intervention groups, and it was not significant for any of the outcome measures (*p* > 0.05). The longitudinal analyses were adjusted for age, sex, BMI and baseline values of the outcome measures. The category ‘no attempts’ was considered as the reference category in longitudinal analyses.

## Results

Table 1 shows baseline associations of the studied outcomes and weight loss history. Altogether, 84% had attempted to lose weight during their lifetime, and most often reported doing so for three times or more (41%). Only few (4%) reported no conscious efforts to manage their weight, which is neither attempting to lose weight nor trying to keep their weight stable. Participants who reported trying to keep their weight stable were the oldest (Table 1). Those who had attempted to lose weight during their lifetime, especially more frequently, were more likely to be female, and to have higher education level, higher risk of developing T2D based on the FINDRISC score, greater BMI, larger waist circumference and more emotional eating than those who had either no previous weight loss attempts or had been trying to keep their weight stable. Fasting plasma insulin concentration was the highest in the category ‘no attempts’. The results separately for women and men are presented in the supplementary materials (Supplementary Tables 1 and 2).

**Table 1:** Baseline associations of anthropometric, metabolic, psychological, and lifestyle outcomes with weight loss history (*n* = 2684).

Variable	Total	Number of prior weight loss attempts					*P*
		A	B	C	D	E	
		No attempts (*n* = 101)	No attempts to lose weight, but trying to keep weight stable (*n* = 332)	1–2 attempts (*n* = 508)	≥3 attempts (*n* = 1094)	Continuously (*n* = 649)	
Proportion of participants, %	100	3.8	12.4	18.9	40.8	24.2	–
Age, years	55.3 (9.7)	56.3 (9.7)_B_	58.8 (9.0)_A,C, D, E_	55.1 (9.4)_B_	54.4 (9.6)_B_	55.1 (10.1)_B_	<**0.001**
Females (%)	2150 (80)	65 (64)	218 (65)	394 (77)	920 (84)	553 (85)	**<0.001**
Education							
Basic	201 (7.5)	12 (11.9)	35 (10.5)	39 (7.7)	62 (5.7)	53 (8.2)	
Middle	729 (27.2)	30 (29.7)	103 (31.0)	135 (26.6)	288 (26.3)	173 (26.7)	
High	1754 (65.4)	59 (58.4)	194 (58.4)	334 (65.7)	744 (68.0)	423 (65.2)	**0.016**
FINDRISC	16 (3.3)	14.9 (3.8)_C,D,E_	14.9 (3.0)_C,D,E_	15.8 (3.2)_A,B,D,E_	16.4 (3.3)_A,B,C_	16.6 (3.3)_A,B,C_	**0.009**
BMI, kg/m^2^	31.2 (5.3)	27.5 (4.9)_C,D,E_	26.6 (3.3)_C,D,E_	30.2 (5.0)_A,B,D,E_	32.3 (5.1)_A,B,C,E_	32.9 (5.2)_A,B,C,D_	<**0.001**
Waist circumference, cm	102 (12.9)	95.3 (13.4)	93.4 (10.7)_C,D,E_	100.4 (13.0)_B_	104.1 (12.3)_B_	105 (12.2)_B_	**0.004**
Systolic blood pressure, mmHg	140.3 (17.5)	142.7 (18.5)_C,E_	143.2 (19.4)_C,D,E_	138.7 (17.0)_A,B_	140 (16.8)_B_	140.1 (17.7)_A,B_	**0.018**
Diastolic blood pressure, mmHg	88.2 (9.6)	88.6 (10.3)	87 (9.1)	87.3 (9.5)	88.8 (9.6)	88.3 (9.8)	0.395
Fasting blood glucose (mmol/l)	5.6 (0.5)	5.7 (0.5)	5.6 (0.5)	5.6 (0.6)	5.6 (0.5)	5.7 (0.5)	0.248
2h-blood glucose (mmol/l)	6.4 (1.7)	6.6 (1.5)	6.2 (1.7)	6.4 (1.8)	6.4 (1.7)	6.4 (1.7)	0.218
HbA1c (mmol/mol)	36 (4)	37 (4)	36 (4)	36 (4)	37 (4)	36 (4)	0.295
Fasting plasma insulin, pmol/l	87 (68)	113 (218)_B,C,D,E_	65 (42)_A_	82 (51)_A_	89 (55)_A_	95 (61)_A_	<**0.001**
2h-plasma insulin (pmol/l)	583 (576)	577 (535)	470 (429)	590 (579)	597 (619)	611 (562)	0.256
Matsuda insulin sensitivity index	12 (8)	13 (9)	15 (9)	13 (8)	12 (7)	11 (8)	0.456
Disposition index	429 (203)	402 (161)	438 (194)	429 (212)	433 (203)	421 (207)	0.204
Total cholesterol (mmol/l)	5.19 (0.98)	5.27 (1.0)	5.25 (0.99)	5.13 (0.90)	5.21 (0.99)	5.15 (0.98)	0.413
LDL cholesterol (mmol/l)	3.19 (0.86)	3.18 (0.92)	3.20 (0.89)	3.18 (0.80)	3.20 (0.86)	3.19 (0.86)	0.852
HDL cholesterol (mmol/l)	1.52 (0.39)	1.59 (0.46)	1.59 (0.44)	1.49 (0.39)	1.52 (0.38)	1.50 (0.37)	0.128
Triglycerides (mmol/l)	1.40 (0.73)	1.43 (0.75)	1.29 (0.61)	1.40 (0.72)	1.47 (0.82)	1.46 (0.76)	0.440
Emotional eating^a^	51 (30)	34 (32)_C,D,E_	31 (23)_C,D,E_	46 (28)_A,B,D,E_	56 (28)_A,B,C_	59 (30)_A,B,C_	<**0.001**
Nutrition self-efficacy, total^b^	2.8 (0.5)	2.8 (0.4)	2.9 (0.4)	2.9 (0.5)	2.8 (0.5)	2.8 (0.5)	0.413
Emotional nutrition self-efficacy^c^	2.6 (0.6)	2.6 (0.5)	2.8 (0.6)	2.7 (0.6)_E_	2.6 (0.6)	2.6 (0.7)_C_	**0.047**
Social nutrition self-efficacy^d^	3 (0.5)	3 (0.4)	3 (0.4)	3 (0.4)	3 (0.5)	3 (0.5)	0.755
Healthy Diet Index^e^	63 (11)	63 (11)	63 (11)	62 (11)	63 (11)	63 (11)	0.566

Values are reported as mean (SD) for continuous outcomes and as frequency (%) for categorical outcomes. *p*-values in bold show significant difference in the outcome measure between the categories of weight loss history. Analysis of Covariance (ANCOVA) was used for continuous outcomes and Chi-squared test was used for categorical outcomes. Each category of weight loss history is designated with an upper-case letter above its column. These letters, as in subscripted form within the table cells, indicate significant pairwise differences. For example, mean age of the category A (No attempts) is significantly different from that of the category B (No attempts to lose weight, but trying to keep weight stable). Age analysis was adjusted for sex. BMI analysis was adjusted for age and sex. All other outcomes were adjusted for age, sex, and BMI. Systolic and diastolic blood pressures were additionally adjusted for taking blood pressure medication, *n* = 1910. Lipid outcomes were additionally adjusted for taking lipid-lowering medication, *n* = 1899; HbA1c, *n* = 2661; Fasting and 2h-plasma insulin, *n* = 2585; Matsuda index and Disposition index, *n* = 2569.

*FINDRISC* Finnish Diabetes Risk Score, *BMI* Body Mass Index, *2* *h-blood glucose* blood glucose 2 h following ingestion of 75 g oral glucose, *HbA1c* glycated hemoglobin, *2* *h-plasma insulin* plasma insulin 2 h following ingestion of 75 g oral glucose, *LDL* low-density lipoprotein, *HDL* high-density lipoprotein.

^a^Score range = 0–12.

^b,c,d^Score range = 1–4.

^e^Score range = 0–100.

**Fig. 1:**
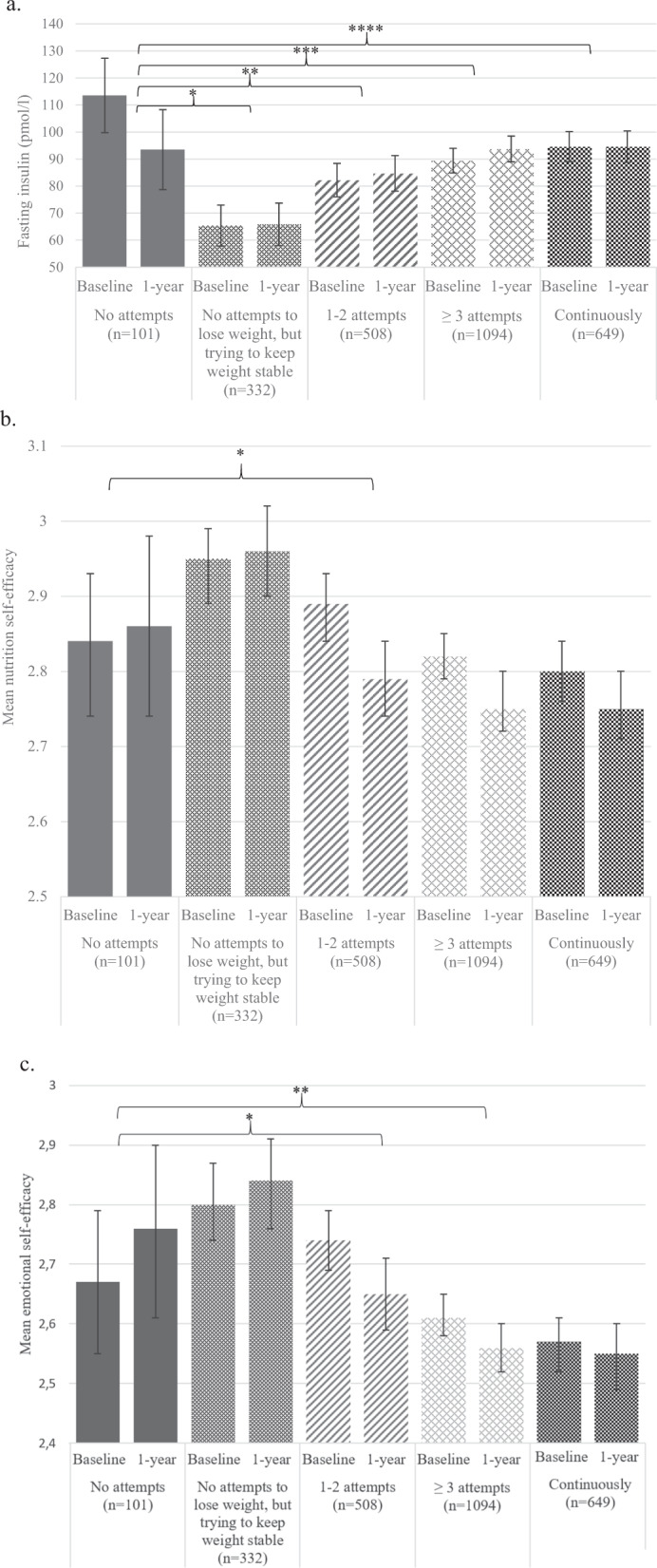
Baseline and 1-year levels of the three outcome measures, for which 1-year changes were significantly different between the categories of weight loss history with ‘no attempts’ as the reference category. **a** Baseline and 1-year levels of fasting plasma insulin concentration in categories of weight loss history (*p* = 0.003, linear mixed-effect model), adjusted for age, sex, baseline body mass index, baseline fasting plasma insulin, and intervention group assignment. *p = 0.002, **p = 0.001, ***p < 0.001, ****p = 0.002. **b** Baseline and 1-year levels of total nutrition self-efficacy (possible score range 1–4) in categories of weight loss history (*p* = 0.02, linear mixed-effect model), adjusted for age, sex, baseline body mass index, baseline total nutrition self-efficacy, and intervention group assignment. *p = 0.05. **c** Baseline and 1-year levels of emotional nutrition self-efficacy (possible score range 1–4) in categories of weight loss history (*p* = 0.014, linear mixed-effect model), adjusted for age, sex, baseline body mass index, baseline emotional nutrition self-efficacy, and intervention group assignment. *p = 0.018, **p = 0.046.

Among all the outcomes tested for (see section 2.2 and Table 1), weight loss history showed a significant contribution to 1-year changes in fasting plasma insulin (*p* = 0.003), total nutrition self-efficacy (*p* = 0.02) and emotional nutrition self-efficacy (*p* = 0.014), regardless of the intervention group assignment (Fig. 1). Mean fasting plasma insulin concentration (Fig. 1a) decreased in individuals with no previous weight loss attempts [−20 pmol/l (−17.6%)] and this change was significantly different from all the other categories of weight loss history, i.e. ‘no attempts to lose weight, but trying to keep weight stable’ [+0.5 pmol/l (+0.7%)], ‘1-2 attempts’ [+2.4 pmol/l (+2.9%)], ‘3 or more attempts’ (+4.3 pmol/l (+4.8%)] and ‘continuously' (−0.03 pmol/l (−0.03%)] (*p* = 0.002, *p* = 0.001, *p* < 0.001, *p* = 0.002, respectively). Slight increases in total nutrition self-efficacy and emotional nutrition self-efficacy were observed in individuals who had no previous weight loss attempts, whereas those with at least one previous weight loss attempt experienced a decrease in both measures. For total nutrition self-efficacy (Fig. 1b), the categories ‘no attempts’ and ‘1-2 attempts’ differed from each other [+0.02 (+0.7%) vs. −0.1 (−3.4%), *p* = 0.05]. Changes in emotional nutrition self-efficacy (Fig. 1c) were significantly different between ‘no attempts’ vs. ‘1-2 attempts’ [+0.09 (+3.3%) vs. −0.09 (−3.3%), *p* = 0.018], and between ‘no attempts’ vs. ‘3 or more attempts’ [+0.09 (+3.3%) vs. −0.05 (−1.9%), *p* = 0.046]. No other significant changes in outcome measures were observed in the categories of weight loss history. The intervention group assignment did not affect the results.

## Discussion

In the present study population, almost all participants had consciously tried to manage their weight during their lifetime. Those who had attempted to lose weight, in particular more frequently, were more likely to have unfavourable characteristics related to T2D, compared to those who had either no previous weight loss attempts or who had been trying to keep their weight stable. Interestingly, those reporting no previous weight loss attempts had the highest initial fasting plasma insulin concentration but also the greatest improvement in this measure. Furthermore, their total and emotional nutrition self-efficacy slightly improved, whereas these measures rather decreased in the other categories. These findings were independent of the intervention group assignment.

In assessing weight loss history, we aimed at distinguishing those who had previous conscious effort for weight management (trying to keep weight stable/trying to lose weight) from those who had no conscious efforts (no attempts for weight loss/maintenance). Almost all participants (96%) reported having consciously tried to manage their weight during their lifetime; either attempting to lose weight (84%) or trying to keep their weight stable (12%). In other words, only a very small proportion of participants (4%) had not made any conscious efforts for weight management. This is in line with previous findings showing that attempts to lose weight are very common among adults in general populations (42%) [5] and especially among individuals with T2D (71%) [51]. With these very high levels of ongoing individual efforts to lose weight, and weight loss being recommended for people at risk or diagnosed with T2D in order to improve glycemic control, it is of high importance to understand the consequences of frequent attempts to lose weight.

In the present study, attempts to lose weight were more common among women than among men. This could be partially attributed to higher health-consciousness and greater interest in healthy lifestyle among women [52]. However, this finding could be biased due to significantly more women participating in the RCT. In addition, having more previous weight loss attempts was most common among those having high education levels, which is also consistent with previous findings [5, 53, 54]. Individuals with higher education levels may attempt to lose weight more frequently, partially resulting from higher awareness of consequences of obesity and the social norm of lower body weight as well as better accessibility of services due to higher socioeconomic status [55, 56].

Participants with previous weight loss attempts showed higher baseline BMI, and larger waist circumference, particularly compared to those who had been trying to keep their weight stable. It may be suggested that attempting to lose weight, especially more frequently, may eventually result in increased total and central adiposity. Similar findings have been reported in some previous cross-sectional and prospective studies [11, 57]. More attempts to lose weight may lead to weight fluctuation, subsequent increase in abdominal obesity [11, 58, 59] and insulin resistance [60]. This pathway could be plausible also in our study population, although weight fluctuation in terms of weight loss magnitude was not studied. However, we could not draw any cause-effect relationship due to cross-sectional nature of the analysis, as individuals with more obesity/abdominal obesity may attempt to lose weight more frequently. In addition, higher FINDRISC score with more prior weight loss attempts in the present study could denote a need for healthier lifestyle adoption by these individuals, such as increasing fiber intake and being more physically active [31]. A few studies investigating the association between prior intentional attempts to lose weight and diabetes-related outcomes and risk factors have in fact yielded inconsistent findings [8, 11, 54, 61, 62], probably due to the use of different weight loss methods or different characteristics of study participants.

We found that mean fasting plasma insulin was significantly higher among those with no prior weight loss attempts than in the other weight loss attempt categories. However, after one year, mean fasting insulin concentration noticeably decreased in the ‘no attempts’ category, whereas only slight changes occurred in the other categories. This finding was independent of initial BMI, waist circumference and fasting insulin concentration, suggesting that individuals with no prior attempts to lose weight could benefit the most from incorporating modifications into their lifestyle. Although StopDia was not a weight loss intervention, those with no prior weight loss attempts being more motivated to implement a set of lifestyle changes and higher compliance with the program may partly justify this finding. On the other hand, as no beneficial changes were observed in those with any number of prior weight loss attempts, this finding gives additional evidence for probable negative metabolic consequences of repeated weight loss attempts. Previous studies have similarly reported a negative association between history of repeated weight loss attempts and adherence to lifestyle modification programs as well as poorer long-term weight loss outcomes [24, 63, 64].

Factors underlying frequent attempts to lose weight and its association with unfavourable metabolic changes are not clear; however, a physiological response to weight loss by means of restoring fat reserves and weight cycling have been suggested as potential contributors [54, 65, 66]. Repeated dieting and weight cycling have been reported to have negative health consequences, particularly in normal-weight individuals [11, 67]. Overall, it seems that unnecessary weight loss attempts should be avoided, and in any necessity to lose weight proper strategies to support maintenance of the reduced weight should be guaranteed.

Interestingly, after the 1-year lifestyle intervention, those who had previously attempted to lose weight, even once, experienced a small decrease in nutrition self-efficacy in general and in emotional nutrition self-efficacy in particular. On the contrary, those who had no prior weight loss attempts experienced a slight increase in these measures. However, it should be noted that the changes in nutrition self-efficacy measures were small, and we cannot conclude their clinical significance. This is, however, an interesting finding and warrants further research on whether even few attempts to lose weight could disadvantageously affect self-efficacy. Furthermore, it probably suggests that the StopDia intervention was not adequately effective for improving nutrition self-efficacy among those with prior weight loss attempts. Therefore, a more targeted approach for improving nutrition self-efficacy, e.g. through identifying reasons behind the previous failures in losing weight should be considered in future lifestyle interventions involving similar target populations. From the perspective of habit theory, this group may benefit more from habit-breaking skills, which demands more effortful strategies than forming a new habit [68].

Those with greater number of weight loss attempts reported more initial emotional eating, although longitudinal changes in this measure were not significant. Similarly, previous studies have reported more emotional eating and more disinhibition of eating among individuals with previous weight loss attempts compared to those with no previous attempts [39, 69]. Thus, as participants in the present study were at high risk for T2D and emotional eating has been linked to unfavourable metabolic characteristics [19, 70, 71], we suggest that future T2D prevention and treatment programs should address emotional eating, particularly among individuals with a history of repeated attempts to lose weight.

As for diet quality, no baseline and longitudinal differences between the categories of weight loss attempt history were observed. This finding contradicts the findings of a recent study among the general Finnish population, which found that individuals with previous intentional weight loss attempts had a better diet quality than those with no previous attempts [54]. On the other hand, individuals with prior intentional weight loss attempts and the lowest diet quality had a greater 15-year T2D risk, compared to those with the lowest diet quality and no prior weight loss attempts [54]. Different measures for self-reported diet quality and different characteristics of the study populations could justify discrepancy of these findings. However, further research is needed to better understand plausible interactions of weight loss attempt history and diet quality regarding T2D risk.

### Strengths and limitations

Our study has various strengths such as a large study sample as well as using a comprehensive set of anthropometric, clinical, metabolic, lifestyle, and behavioural factors related to T2D risk, which to our knowledge is the first of its kind. The FINDRISC was used to reach individuals at risk for T2D that is a simple validated non-invasive tool. The lifestyle intervention was integrated into the routines in health care centers, which could decrease burden of committing and participating in the intervention.

As a limitation, women comprised 80% of the participants in the present study, which could limit generalizability of the findings. However, ‘sex’ was adjusted for in all the statistical analyses. For the weight loss history, only number of previous attempts to lose weight was inquired, but not the net weight change (net increase/decrease) or the amount of weight change. Thus, we could not study whether weight fluctuation was modifying the effect of weight loss history on the outcomes. Another important limitation of the study was that the number of participants in the ‘no attempts’ category was less than in the other categories, which might affect the comparisons between the weight loss history categories. Furthermore, participants in the control group received brief recommendations for diet and physical activity, which could at least partially explain why the intervention group assignment had no effects on the results. Finally, it should be noted that as the present study was a secondary analysis of the StopDia intervention study, the analyses were not considered in the power calculation.

## Conclusions

Great majority of individuals at risk for T2D, who participated in the Stop Diabetes T2D prevention intervention had attempted to lose weight during their lifetime. Particularly those with more frequent attempts to lose weight had less favourable profile for several T2D risk factors. Individuals with no previous weight loss attempts particularly benefited from participating in the StopDia study, showing slight improvements in fasting insulin as well as in nutrition self-efficacy. These findings suggest that multiple attempts to lose weight may unfavourably affect T2D risk factors as well as efficacy of lifestyle modification. Therefore, we suggest that frequency of prior weight loss attempts should be taken into account for designing T2D prevention strategies, although more research is needed on the effects of conscious weight loss efforts on T2D risk factors.

## Supplementary information

Supplemetary material
