# Supplementary material for: Associations between weight loss history and factors related to type 2 diabetes risk in the Stop Diabetes study
Source: Int J Obes (Lond). 2022 Jan 12;46(5):935–42. doi: 10.1038/s41366-021-01061-4 (PMC9050593; doi:10.1038/s41366-021-01061-4)
Supplement: Supplementary file 1 — Supplemetary material [file 41366_2021_1061_MOESM1_ESM.docx]

Supplementary Table 1. Baseline associations of anthropometric, metabolic, psychological, and lifestyle outcomes with weight loss history in women (n=2150)

| Variable | Number of prior weight loss attempts | | | | |  |
| --- | --- | --- | --- | --- | --- | --- |
|  | **A** | **B** | **C** | **D** | **E** | *P* |
|  | No attempts | No attempts to lose weight, but trying to keep weight stable | 1-2 attempts | ≥ 3 attempts | Continuously |  |
|  | (n=65) | (n=218) | (n=394) | (n=920) | (n=553) |  |
| Proportion, % | 3.0 | 10.1 | 18.3 | 42.8 | 25.7 | - |
| Age, years | 55.5 (8.9) | 58.2 (9.6)**_C,D, E_** | 54.7 (9.6)**_B_** | 54.3 (9.7)**_B_** | 54.7 (10.2)**_B_** | **< 0.001** |
| Education |  |  |  |  |  |  |
| Basic | 7 (10.8) | 26 (11.9) | 31 (7.9) | 51 (5.5) | 43 (7.8) |  |
| Middle | 14 (21.5) | 61 (28.0) | 97 (24.6) | 237 (25.8) | 142 (25.7) |  |
| High | 44 (67.7) | 131 (60.1) | 266 (67.5) | 632 (68.7) | 368 (66.5) | 0.066 |
| FINDRISC | 14.8 (3.7)**_D,E_** | 15.0 (2.9)**_D,E_** | 15.8 (3.2) | 16.4 (3.2)**_A,B_** | 16.6 (3.3)**_A,B_** | **0.005** |
| BMI, kg/m^2^ | 26.6 (5.0)**_C,D,E_** | 26.1 (3.4)**_C,D,E_** | 29.9 (5.0)**_A,B,D,E_** | 32.3 (5.2)**_A,B,C,E_** | 32.9 (5.4) **_A,B,C,D_** | **< 0.001** |
| Waist circumference, cm | 90.8 (12.5) | 89.4 (9.8) | 97.6 (12.0) | 102.6 (12.0) | 103.4 (11.6) | 0.137 |
| Systolic blood pressure, mmHg | 139.5 (17.6) | 141.5 (19.6) | 137.3 (17.5) | 139 (16.7) | 138.9 (17.5) | 0.053 |
| Diastolic blood pressure, mmHg | 87.5 (10.3) | 86.4 (8.7) | 87 (9.3) | 88.2 (9.3) | 87.9 (9.7) | 0.695 |
| Fasting blood glucose (mmol/l) | 5.5 (0.4) | 5.5 (0.5) | 5.5 (0.6) | 5.5 (0.5) | 5.6 (0.5) | 0.374 |
| 2h-blood glucose (mmol/l) | 6.5 (1.5) | 6.0 (1.7) | 6.3 (1.8) | 6.4 (1.7) | 6.3 (1.7) | 0.198 |
| HbA1c (mmol/mol) | 35.9 (4) | 36.2 (3.6) | 35.9 (4.1) | 35.8 (3.9) | 36.3 (4) | 0.329 |
| Fasting plasma insulin, pmol/l | 84.7 (144.7)**_B,C,D,E_** | 62.2 (42.6)**_A_** | 76.4 (45.6)**_A_** | 85.3 (50.7)**_A_** | 91.2 (59.3)**_A_** | **0.006** |
| 2h-plasma insulin (pmol/l) | 497.6 (378.9) | 457.4 (434.9) | 540.4 (458.8) | 566.6 (558.2) | 600.6 (567.7) | 0.523 |
| Matsuda insulin sensitivity index | 15 (9.7) | 16.1 (8.9) | 13.6 (8) | 12.4 (7.6) | 11.7 (7.8) | 0.679 |
| Disposition index | 438 (162.9) | 470.2 (203.8) | 450.3 (223.6) | 446.5 (207.6) | 431.8 (212.3) | 0.281 |
| Total cholesterol (mmol/l) | 5.5 (1) | 5.4 (1) | 5.2 (0.9) | 5.2 (1) | 5.2 (1) | 0.327 |
| LDL cholesterol (mmol/l) | 3.3 (0.9) | 3.2 (0.9) | 3.2 (0.8) | 3.2 (0.8) | 3.2 (0.9) | 0.845 |
| HDL cholesterol (mmol/l) | 1.7 (0.5) | 1.7 (0.4) | 1.6 (0.4) | 1.6 (0.4) | 1.5 (0.3) | 0.207 |
| Triglycerides (mmol/l) | 1.4 (0.8) | 1.2 (0.5) | 1.3 (0.7) | 1.4 (0.6) | 1.4 (0.7) | 0.375 |
| Emotional eating^a^ | 37.8 (31.3)**_C,D,E_** | 34.3 (23.2)**_C,D,E_** | 49 (27.6)**_A,B,D,E_** | 59 (27.5)**_A,B,C,E_** | 62.9 (28)**_A,B,C,D_** | **< 0.001** |
| Nutrition self-efficacy, total^b^ | 2.8 (0.5) | 2.9 (0.4) | 2.9 (0.5)_D,E_ | 2.8 (0.5)_C_ | 2.8 (0.5)_C_ | **0.01** |
| Emotional nutrition self-efficacy^c^ | 2.6 (0.6)_C_ | 2.7 (0.5)_E_ | 2.7 (0.6)_A,D,_**_E_** | 2.6 (0.6)_C_ | 2.5 (0.7)_B,_**_C_** | **< 0.001** |
| Social nutrition self-efficacy^d^ | 3 (0.4) | 3 (0.4) | 3 (0.4) | 3 (0.5) | 3 (0.5) | 0.625 |
| Healthy Diet Index^e^ | 63 (11.6) | 65.6 (10.5) | 61.9 (11) | 62.7 (10.6) | 63.1 (11) | 0.105 |

NOTE.– Values are reported as mean (SD) for continuous outcomes and as frequency (%) for categorical outcomes. Analysis of Covariance (ANCOVA) was used for continuous outcomes and Chi-squared test was used for categorical outcomes. Each category of weight loss history is designated with an upper-case letter above its column. These letters, as in subscripted form within the table cells, indicate significant pairwise differences. For example, mean age of the category C (1-2 attempts) is significantly different from that of the category B (No attempts to lose weight, but trying to keep weight stable). FINDRISC: Finnish Diabetes Risk Score; BMI: Body Mass Index; 2h-blood glucose: blood glucose 2 hours following ingestion of 75g oral glucose; HbA1c: glycated hemoglobin; 2h-plasma insulin: plasma insulin 2 hours following ingestion of 75g oral glucose; LDL: low-density lipoprotein; HDL: high-density lipoprotein. Age analysis was adjusted for sex. BMI analysis was adjusted for age and sex. All other outcomes were adjusted for age, sex, and BMI. Systolic and diastolic blood pressures were additionally adjusted for taking blood pressure medication, n=1536. Lipid outcomes were additionally adjusted for taking lipid-lowering medication, n=1531; HbA1c, n=2140; Fasting plasma insulin, n=2084; 2h-plasma insulin, n=2078; Matsuda index and Disposition index, n=2071; Healthy Diet Index, n=2118.

^a^ Score range = 0-12, ^b,c,d^ Score range = 1-4, ^e^ Score range = 0-100

Supplementary Table 2. Baseline associations of anthropometric, metabolic, psychological, and lifestyle outcomes with weight loss history in men (n=534)

| Variable | Number of prior weight loss attempts | | | | |  |
| --- | --- | --- | --- | --- | --- | --- |
|  | **A** | **B** | **C** | **D** | **E** | *P* |
|  | No attempts | No attempts to lose weight, but trying to keep weight stable | 1-2 attempts | ≥ 3 attempts | Continuously |  |
|  | (n=36) | (n=114) | (n=114) | (n=174) | (n=96) |  |
| Proportion, % | 6.7 | 21.3 | 21.3 | 32.6 | 18.0 | - |
| Age, years | 57.8 ( 11.2) | 60 (7.9)**_C,D, E_** | 56.6 (9.0)**_B_** | 55.1 (9.0)**_B,E_** | 57.5 (9.2)**_B,D_** | **< 0.001** |
| Education |  |  |  |  |  |  |
| Basic | 5 (13.9) | 9 (7.9) | 8 (7.0) | 11 (6.3) | 10 (10.4) |  |
| Middle | 16 (44.4) | 42 (36.8) | 38 (33.3) | 51 (29.3) | 31 (32.2) |  |
| High | 15 (41.7) | 63 (55.3) | 68 (59.6) | 112 (64.4) | 55 (57.3) | 0.374 |
| FINDRISC | 15.0 (4.0) | 14.7 (3.4)**_D,E_** | 15.8 (3.1) | 16.2 (3.4)**_B_** | 16.7 (3.4)**_B_** | 0.052 |
| BMI, kg/m^2^ | 29.0 (4.4)**_C,D,E_** | 27.8 (3.0)**_C,D,E_** | 31.5 (4.9)**_A,B,E_** | 32.3 (4.6)**_A,B,E_** | 33.0 (4.4) **_A,B,C_** | **< 0.001** |
| Waist circumference, cm | 103.7 (11.2) | 101.0 (8.1)**_C,D,E_** | 110.5 (11.7)**_B_** | 112.3 (11.0)**_B_** | 114.6 (11.5)**_B_** | **0.05** |
| Systolic blood pressure, mmHg | 150 (19.1) | 146.2 (19) | 144 (14.1) | 145.7 (17) | 146.8 (17.4) | 0.337 |
| Diastolic blood pressure, mmHg | 91 (10.2) | 88.2 (9.9) | 88.7 (10.4) | 91.8 (11) | 91 (10.3) | 0.332 |
| Fasting blood glucose (mmol/l) | 6.0 (0.5) | 5.8 (0.5) | 5.9 (0.5) | 5.9 (0.5) | 5.9 (0.5) | 0.31 |
| 2h-blood glucose (mmol/l) | 6.8 (1.6) | 6.5 (1.8) | 6.7 (1.9) | 6.8 (1.9) | 6.9 (1.7) | 0.936 |
| HbA1c (mmol/mol) | 38.4 (3.9) | 36.9 (3.7) | 37.3 (3.7) | 36.9 (3.8) | 36.5 (3.7) | 0.075 |
| Fasting plasma insulin, pmol/l | 164.3 (304.7)**_B,C,D,E_** | 71.7 (41.5)**_A_** | 102.8 (63.4)**_A_** | 112.7 (71.5)**_A_** | 115.4 (70.1)**_A_** | **< 0.001** |
| 2h-plasma insulin (pmol/l) | 716.2 (720.3) | 495.9 (419.7) | 765.9 (864) | 769.3 (865) | 670.9 (527.8) | 0.226 |
| Matsuda insulin sensitivity index | 10.6 (7.7) | 13.5 (8) | 9.8 (6.5) | 9.5 (6.4) | 9 (5.9) | 0.497 |
| Disposition index | 340.3 (142.3) | 377 (157.9) | 352.5 (140.8) | 361.3 (154.7) | 355.6 (154.2) | 0.721 |
| Total cholesterol (mmol/l) | 4.6 (0.9) | 5 (1) | 4.7 (1) | 5 (1) | 4.9 (0.9) | 0.396 |
| LDL cholesterol (mmol/l) | 2.8 (0.7) | 3.1 (0.9) | 3 (0.9) | 3 (0.9) | 3.1 (0.8) | 0.37 |
| HDL cholesterol (mmol/l) | 1.4 (0.4) | 1.3 (0.4) | 1.2 (0.3) | 1.3 (0.3) | 1.3 (0.3) | 0.495 |
| Triglycerides (mmol/l) | 1.4 (0.7) | 1.4 (0.7) | 1.6 (0.9) | 1.8 (1.4) | 1.6 (0.8) | 0.787 |
| Emotional eating^a^ | 21.6 (25)**_C,D,E_** | 23.9 (22.1)**_D,E_** | 34.9 (23.7)**_A_** | 39.5 (26.1)**_A,B_** | 41.8 (30.7)**_A,B_** | **0.001** |
| Nutrition self-efficacy, total^b^ | 2.9 (0.5) | 3 (0.5) | 2.8 (0.4) | 2.9 (0.5) | 3 (0.5) | 0.345 |
| Emotional nutrition self-efficacy^c^ | 2.8 (0.6) | 2.9 (0.6) | 2.8 (0.5) | 2.8 (0.6) | 2.8 (0.7) | 0.496 |
| Social nutrition self-efficacy^d^ | 3 (0.4) | 3 (0.4)_C_ | 2.9 (0.4)_B,E_ | 3 (0.5) | 3 (0.4)_C_ | **0.027** |
| Healthy Diet Index^e^ | 59.3 (10) | 60 (9.9) | 58.1 (11) | 58.3 (10.9) | 60.9 (9.9) | 0.326 |

NOTE.– Values are reported as mean (SD) for continuous outcomes and as frequency (%) for categorical outcomes. Analysis of Covariance (ANCOVA) was used for continuous outcomes and Chi-squared test was used for categorical outcomes. Each category of weight loss history is designated with an upper-case letter above its column. These letters, as in subscripted form within the table cells, indicate significant pairwise differences. For example, mean age of the category C (1-2 attempts) is significantly different from that of the category B (No attempts to lose weight, but trying to keep weight stable). FINDRISC: Finnish Diabetes Risk Score; BMI: Body Mass Index; 2h-blood glucose: blood glucose 2 hours following ingestion of 75g oral glucose; HbA1c: glycated hemoglobin; 2h-plasma insulin: plasma insulin 2 hours following ingestion of 75g oral glucose; LDL: low-density lipoprotein; HDL: high-density lipoprotein. Age analysis was adjusted for sex. BMI analysis was adjusted for age and sex. All other outcomes were adjusted for age, sex, and BMI. Systolic and diastolic blood pressures were additionally adjusted for taking blood pressure medication, n=374. Lipid outcomes were additionally adjusted for taking lipid-lowering medication, n=368; HbA1c, n=521; Fasting plasma insulin, n=501; 2h-plasma insulin, n=500; Matsuda index and Disposition index, n=498; Healthy Diet Index, n=532.

^a^ Score range = 0-12, ^b,c,d^ Score range = 1-4, ^e^ Score range = 0-100
